# Generalized expectancy of threat in threatening compared to safe contexts

**DOI:** 10.1093/scan/nsae097

**Published:** 2024-12-16

**Authors:** Asimina Aslanidou, Marta Andreatta, Alex H.K Wong, Matthias J Wieser

**Affiliations:** Department of Psychology, Education and Child Studies, Erasmus University Rotterdam, Rotterdam 3000 DR, The Netherlands; Department of General Psychiatry and Psychotherapy, University Hospital Tübingen, Tübingen, D-72076, Germany; Department of Psychology, Education and Child Studies, Erasmus University Rotterdam, Rotterdam 3000 DR, The Netherlands; Department of Psychology, Education and Child Studies, Erasmus University Rotterdam, Rotterdam 3000 DR, The Netherlands

**Keywords:** fear generalization, cue-in-context, steady-state visual evoked potentials, skin conductance, context

## Abstract

Fear of threatening contexts often generalizes to similar safe contexts, but few studies have investigated how contextual information influences cue generalization. In this study, we explored whether fear responses to cues would generalize more broadly in a threatening compared to a safe context. Forty-seven participants underwent a differential cue-in-context conditioning protocol followed by a generalization test, while we recorded psychophysiological and subjective responses. Two faces appeared on a computer screen in two contexts. One face (CS+) in the threat context (CTX+) was followed by a female scream 80% of the time, while another face (CS−) was not reinforced. No faces were reinforced in the safe context (CTX−). In the generalization test, the CSs and four morphs varying in similarity with the CS+ were presented in both contexts. During acquisition, conditioned responses to the cues were registered for all measures and the differential responding between CS+ and CS− was higher in CTX+ for US-expectancy ratings and skin conductance responses, but the affective ratings and steady-state visual evoked potentials were not context-sensitive. During test, adaptive generalized responses were evident for all measures. Despite increased US-expectancy ratings in CTX+, participants exhibited similar cue generalization in both contexts, suggesting that threatening contexts do not influence cue generalization.

Generalization of conditioned fear is the transfer of a defensive response associated with a cue that signals danger to innocuous novel stimuli. These stimuli have never been associated with danger, but they might share perceptual or conceptual similarities with the threat-associated cue (Dymond et al. 2015). This transfer can be rather automatic, a process that is very helpful in a dynamic, ever-changing environment, making fear generalization evolutionary relevant. However, overgeneralization of fear can lead to excessive defensive responses to false alarms and excessive avoidance behaviors, which are often found in clinical anxiety (Cooper et al. 2022).

Fear generalization is often studied with a differential fear conditioning paradigm followed by a generalization test (Lonsdorf et al. 2017). In this paradigm, one cue (conditioned stimulus, CS+) is associated with an aversive unconditioned stimulus (US) and thus signals threat. A second cue (CS−) is never associated with the aversive stimulus and is, thus, associated with the absence of threat. In a subsequent generalization test, a set of new generalized stimuli (GSs) are presented along with the CS+ and CS−, which lie on a continuum of similarity from the CS+ to the CS− (Lissek et al. 2008; Fig. 1). Generalization of conditioned responses has been measured with subjective ratings such as US expectancy and arousal ratings (Lissek et al. 2009, Tinoco-González et al. 2015, Ahrens et al. 2016, Wong and Lovibond 2017, Lemmens et al. 2021), psychophysiological measures such as fear-potentiated startle response and skin conductance (Lissek et al. 2009, 2010, Andreatta et al. 2015, Ahrens et al. 2016, Dunsmoor et al. 2017, Wong and Lovibond 2017, Herzog et al. 2021, Lemmens et al. 2021), and imaging measures such as the blood-oxygen-level-dependent response (Greenberg et al. 2013, Cha et al. 2014) and electrocortical signals (McTeague et al. 2015, Stegmann et al. 2020).

**Figure 1.**
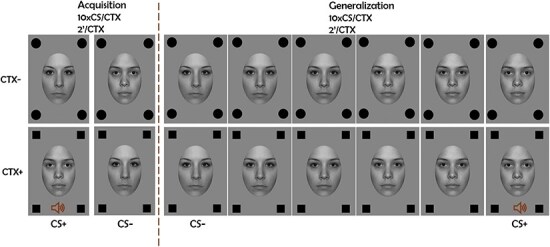
Trial structure in acquisition and generalization.

Preliminary laboratory studies showed that patients with clinical anxiety demonstrate heightened fear responses to a wider range of GSs compared to healthy controls (Lissek et al. 2010, 2014, Kaczkurkin et al. 2017). These findings suggest fear generalization as a transdiagnostic factor that underlies anxiety and stress–related and trauma-related disorders (Cooper et al. 2022). Nonetheless, overgeneralization in anxiety patients or people at risk has been questioned (Torrents-Rodas et al. 2013, Tinoco-González et al. 2015, Ahrens et al. 2016, Lange et al. 2019, Fraunfelter et al. 2022). The inconsistent findings make evident the need for more research into the mechanisms that influence fear generalization before the translation of laboratory findings to clinical practice.

Several external factors have been identified to exert influence on fear generalization. One such factor is contextual information. Self-reported ratings (such as arousal and anxiety ratings) have shown to generalize to contexts that have 50% or higher similarity with a threatening context in which a US was unpredictably presented (CTX+) (Andreatta et al. 2015, 2020, Neueder et al. 2019). The influence of a specific context on fear generalization is of particular interest as using contextual information to predict danger or safety can have a protective effect against overgeneralization of fear (Liberzon and Abelson 2016). However, patients with clinical anxiety seem to show difficulties in inhibiting fear responses in the presence of safety signals and therefore safety learning (Lissek et al. 2005, Duits et al. 2015) and show less differentiation of their responses in a threatening versus a safe context (Steiger et al. 2015).

Learning is inherently linked to the context in which it occurs. During fear conditioning, associations are created not only between the US and the cues but also with contextual information. Context in the conditioning literature comprises both the individual’s internal and external environment in which learning takes place and is defined by its spatial and temporal features (Bouton 2002, Maren et al. 2013). In research, contexts are often created by different visual backgrounds in which stimuli are presented or the surroundings conditioning takes place (Lonsdorf et al. 2017). Emotions or drug-induced changes in internal states can also constitute contexts (Bouton et al. 2006). Context conditioning can occur in any situation a US is presented, and contextual information can determine the degree of fear responses during both fear learning and extinction (Andreatta and Pauli 2021). More specifically, in threatening contexts (contexts in which a US is presented), participants show potentiated fear responses (Baas et al. 2004, Haaker et al. 2013, Lonsdorf et al. 2014, Andreatta et al. 2015), and extinction memories are also thought to be context specific (Kalisch et al. 2006, Battaglia et al. 2018). Moreover, contextual information can modulate the differential responses to cues presented. Several studies have found differences between CS+ and CS− in the context in which a US was present, while no such differences in a safe context and these results have been replicated in both verbal ratings (Andreatta et al. 2020) and physiological responses (for a review, see also Mühlberger et al. 2014, Simon-Kutscher et al. 2019, Andreatta and Pauli 2021).

In contrast to context conditioning, paradigms that allow for comparisons between cues and contexts (cue-in-context paradigms) are considered more ecologically valid than paradigms that include only one or the other as in everyday life, cues and contexts appear intertwined. Moreover, contexts offer important information regarding the nature of a threatening cue (Lonsdorf et al. 2017, Andreatta and Pauli 2021). For instance, a snake encountered while hiking on a mountain signals danger, but a snake in the zoo signals the absence of danger. Importantly, when encountered with ambiguous stimuli, such as GSs, contextual information plays a big role in reducing processing-related ambiguity and in forming flexible and appropriate responses (Maren et al. 2013). However, despite the abundance of studies using a cue-in-context design for acquisition and extinction, there are far fewer studies that use it to investigate cue generalization. With a cue-in-context conditioning paradigm, Klein et al. (2021) investigated the developmental differences between adults and adolescents in threat learning, generalization, and avoidance. During training, participants saw two different rooms with a desk lamp that had either a yellow (CS−) or blue light (CS+). The CS+ was associated with a loud noise US only in the threatening context, while no US was presented in the safe context regardless of the CS type (i.e. a conditional discrimination procedure). In a following generalization phase, different colored lamps along a continuum from yellow to blue in steps of three were presented. The results showed that participants differentiated more between the different stimuli in the threatening context in the danger ratings and generalized their skin conductance responses to more GSs in the threatening than in the safe context. The authors attributed this pattern to the threat ambiguity of the GSs, which might have triggered the participants to rely more on the contextual information. Interestingly, mean danger ratings during generalization positively correlated with trait anxiety and intolerance of uncertainty, but the authors did not examine any context differences in these correlations. However, no other studies, to our knowledge, have further investigated the influence of contextual information in cue generalization.

Additionally, fear generalization is a multilayered phenomenon for which many systems are at play. From cue detection to categorizing it as threat/safe and choosing the appropriate flexible response, the signal passes from different systems, each of which is responsible for a different process. Although most studies employ both psychophysiological and subjective ratings, far less explored fear generalization in early threat detection in the cortical visual system. This can be achieved with the use of electroencephalogram (EEG) and more specifically steady-state visual evoked potentials (ssVEPs). ssVEP is a measure of neuronal activity originating in low-tier visual cortical areas (Di Russo et al. 2007, Wieser et al. 2016a), oscillating at the frequency of a rapidly flickering driving stimulus. Importantly, their amplitudes are not only sensitive to stimulus’ physical characteristics but to more complex processes as well such as working memory, selective attention, and motivational relevance (Müller et al. 1998, Perlstein et al. 2003, Song and Keil 2014) as well as unpredictability of threat (Kastner-Dorn et al. 2018, Stegmann et al. 2023). Previous work in both animals and humans highlights experience-related changes in the visual cortex (Gilbert 1998), and the sustained ssVEP signal has been argued to reflect re-entrant signals from anterior and deeper cortical structures associated with threat such as the amygdala (Damasio 1998, Davis 1998, Miskovic and Keil 2013a). In fear conditioning research, heightened ssVEP amplitudes have been found in response to CS+ in comparison to CS− (Moratti et al. 2006, Miskovic and Keil 2013b, McTeague et al. 2015, Stegmann et al. 2020), which also seems to be influenced by threatening context (Stegmann et al. 2023) and a decrease in response to CS+ after extinction (Miskovic and Keil 2013a). Furthermore, the visual cortex has shown to display a different response pattern to test stimuli compared to the linear or quadratic generalization gradients often found in generalization research (Lissek et al. 2008, 2010, Ahrens et al. 2016). Instead, the lowest response has been observed for the GS closest in similarity to the CS+ displaying a pattern that is thought to reflect the visual cortex effort to discriminate the most motivationally significant stimulus, the CS+, from the rest, similar to lateral inhibition (McTeague et al. 2015, Onat and Büchel 2015, Stegmann et al. 2020). These different generalization gradients (linear/quadratic, lateral inhibition) might reflect the effort of each system involved in fear generalization, to process the threatening stimulus according to their different functions and point to the importance of investigating generalization from different perspectives and with different measures.

As a means of exploring potential mechanisms influencing fear generalization using measures that target different processes, in the present study, we explored the influence of threatening context on cue generalization expressed in different brain and physiological measures as well as affective ratings and threat expectancy. Female faces with neutral facial expressions (cues) presented on gray backgrounds that had a different set of black, geometrical shapes in the corners of the screen (contexts) were presented to participants in a differential fear conditioning paradigm with a generalization test. Measures of ssVEPs, skin conductance response (SCR), and subjective ratings of valence, arousal, and US expectancy were recorded. One of the cues (CS+) predicted the US in only the dangerous context (CTX+) but not in the other, safe context (CTX−). Based on the aforementioned literature, we expected wider generalization gradients for the cues presented in CTX+ compared to CTX− for the measures of SCR and the subjective ratings. Additionally, as the generalization gradients found so far with ssVEPs resemble a pattern of lateral inhibition, we expected the same pattern in CTX+ and no stimulus differentiation in CTX− as the stimuli presented there do not differ in motivational relevance. Finally, we explored whether individual differences such as anxiety and depression traits influence the cue generalization in the different contexts.

## Materials and methods

### Participants

Data were collected from 50(This is a deviation from our preregistration in which we decided to recruit a sample of 40 participants. In light of new results (Aslanidou et al. 2023), we decided to increase our power in order to detect a difference between the CSs in acquisition in ssVEPs if the difference exists as we suspect the effect size to be smaller than the one used for the pre-registration.) undergraduate students at the Erasmus University Rotterdam, in exchange for course credit. *A priori* power analysis using G*power 3.1.9.7, with a small to moderate effect size *f* of 0.20, alpha set at 0.05, and power at 0.85, calculated for the interaction between the CS type and the contexts, indicated a sufficient sample size of 40 participants. Due to equipment failure, three participants were excluded making the final sample 47 (10 males (See the Supplementary data for the main analyses excluding males. There were no differences in the main conclusions.)) with a mean age of 20.84 years (SD* =* 3.05), see also Table 1. Participants with family history of photic epilepsy were excluded from data collection, and all participants had normal or corrected-to-normal vision. The experiment was approved by the Ethical Committee of the Erasmus University Rotterdam and is in accordance with the Declaration of Helsinki. The registration and data of the study can be found in the Open Science Framework (https://doi.org/10.17605/OSF.IO/9ZCEW).

**Table 1.** Sample demographics and means and standard deviations for the questionnaires.

Variable	Count	*M*	SD	Min–Max
Sex	F: 37; M: 10			
*N*	47			
Age, years		20.8	3.1	18–37
BDI-II		11.0	9.5	1.0–33.0
STAI-S		43.5	3.9	35–52
STAI-T		48.2	4.8	38–63
IUS		64.8	18.4	31–97
LSAS		35.5	19.4	1–101

### Materials

#### Stimuli

Two different female faces with a neutral facial expression were used as CSs. The facial stimuli were selected from the NimStim Set of Facial Expressions (03F_NE_C, 10F_NE_C; Tottenham et al. 2009). These stimuli were adjusted for brightness and luminance and converted to gray scale. From these two stimuli, four morphs were created in steps of 20% using face-morphing software (Squirlz Morph; Xiberpix, Solihull, UK), with GS1 being the stimulus closest to the CS+ and GS4 the one closest to CS−. The pictures were presented in two different backgrounds which constituted the two contexts (CTXs). Both CTXs lasted for 2 min and consisted of a gray background (Red Green Blue: 133, 133, 133) and four shapes of the same category (circle or square), one in each corner of the screen, as depicted in Fig. 1. The shapes were black, 100 px each, and differed between CTX+ and CTX−. The type of shapes that represented CTX+ and CTX− was counterbalanced across participants. A female scream with white noise lasting 1 s acted as US and was presented at 90 dB through four free field speakers.

#### Questionnaires

Participants completed four psychometric questionnaires before the experiment. The second edition of Beck’s Depression Inventory (BDI-II; Beck et al. 1961) was used to measure the presence and severity of depressive symptoms, State-Trait Anxiety Inventory (STAI; Spielberger 1970) measured anxiety, the Intolerance of Uncertainty Scale (IUS; Buhr and Dugas 2002) was used to assess the predispositional tendency to find uncertain situations aversive and anxiety provoking (Morriss et al. 2016), and the Liebowitz Social Anxiety Scale (LSAS; Liebowitz 1987) was used to assess the presence of social anxiety symptoms.

#### Ratings

For all ratings, each face (CS+, CS−, and GS1–GS4) was presented once in each context. Participants’ valence and arousal ratings were recorded with the Self-Assessment Manikin Scale (SAM; Bradley and Lang 1994) with a visual scale ranging from 1 “very pleasant” (valence) or “very calm” (arousal) to 9 “very unpleasant” or “very arousing.” Each cue-in-context presentation lasted for 1 s followed by the SAM according to the suggestions of Bradley and Lang (1994). Responses were registered by pressing the arrow keys in the keyboard to indicate the appropriate affective state and then pressing “enter” to confirm their choice. US expectancy was assessed with a visual analog scale and the question “How likely is this face to be followed by a scream?”. This time the cue-in-context presentations were simultaneous to the US-expectancy question and lasted until a response was given (Lonsdorf et al. 2017). Responses were logged by dragging a red bar in the visual analog scale ranging from 0 to 100, to the most appropriate point using the mouse.

A discrimination task was included to monitor participants’ ability to perceptually discriminate the different stimuli (Resnik et al. 2011, Struyf et al. 2017) and whether this ability influences the degree of generalization. The discrimination task included five sets of trials in which participants saw each face again (context was not included in these presentations) presented with the CS+ one by one for 1 s each. After each trial, participants were asked if the two pictures depicted the same face. Responses were recorded using the keyboard and by pressing “y” for yes or “n” for no. The response options remained visible on the screen until a response was given.

#### Study design and procedure

Upon arrival to the laboratory, participants filled in the informed consent and completed the questionnaires. Afterward, they were guided to a separate soundproof room where the EEG and SCR electrodes were attached. Participants were seated in a reclining chair positioned 1.5 m away from the 22-inch iiyama HM204DT-A computer screen which had a 120-Hz refresh rate. They were instructed that they would see some faces and sometimes would hear a loud unpleasant sound and their task was to look at the faces. They received no further instructions regarding the contingencies and the context manipulation. The experiment had a differential cue-in-context conditioning design and included three phases, habituation, acquisition, and generalization. During habituation, the CS+ and CS− were presented once in each context without any US presentation. In acquisition, the CS+ and CS− were presented five times each in each CTX presentation equally, so in total, 10 CS presentations in each CTX. In order to facilitate context learning in the absence of explicit instructions regarding the context contingencies and the small number of CTX trials, CTX+ was always presented first. Each CTX combination was presented twice (40 trials overall: 10× each CS/CTX). One CTX served as CTX+ during which CS+ was reinforced at an 80% rate. The US was presented at the offset of CS+. The CS− was never reinforced. In CTX−, both CSs were presented but not reinforced. After the first presentation of each CTX, participants rated the US expectancy for every cue-in-context presentation. Finally, in the generalization phase, the four GS morphs and the CSs were presented in each CTX presentation, with each stimulus/CTX combination presented 10 times (120 trials: 10× each stimulus/CTX). The CS+ in CTX+ was reinforced 20% similar to a previous experiments from our laboratory (Aslanidou et al. 2023) to minimize premature extinction (Lissek et al. 2008) and promote the emergence of individual differences (Lissek et al. 2006). The remaining stimulus/CTX combinations were not reinforced.

All CS/GS lasted for 5 s and were presented in flickering mode of 15 Hz to evoke ssVEPs. Each CTX presentation lasted for 2 min. The stimuli appeared always together with the CTX and were presented in a pseudorandom order with the restrictions that not more than two presentations of the same face would appear in a row and the acquisition phase would start with CTX+ and CS + . The interstimulus interval (the interval between each stimulus/CTX combination) ranged between 5 and 6 s. Although this interstimulus interval (ISI) is shorter than typically encountered in studies using SCR, this was done to prevent participants’ fatigue especially due to the flickering nature of the stimuli. During the ISI, participants saw the CTX with a fixation cross in the middle of the screen. For a visual description of the trials, see Fig. 1. Participants rated valence and arousal at the end of each phase, US expectancy half-way through and at the end of acquisition, and at the end of generalization. Stimulus discrimination was assessed only at the end of generalization so as to not influence participants’ responses during that phase.

#### Psychophysiological recording and analysis

EEG was recorded from 64 Ag/AgCl active scalp electrodes attached to an elastic cap according to the 10/20 system. A Biosemi ActiveTwo system (Biosemi, Amsterdam, The Netherlands) was used to amplify the signal which was further digitized at a 512 Hz sampling rate with a 24-bit analog-to-digital conversion. The impedance was kept below 30 kΩ. The Biosemi ActiveTwo system includes two extra electrodes instead of a single ground electrode, namely, Common Mode Sense and Driven Right Leg which act as online reference and ground. Horizontal and vertical eye movements were recorded by placing two flat type active electrodes on the two outer canthi of the eye (horizontal electro-ocular activity) and two more in the infraorbital and supraorbital region of the right eye (vertical electro-ocular activity).

Electrodermal activity (EDA) was recorded using two 8-mm Ag/AgCl electrodes with 0.05 M NaCl electrolyte medium and the same Biosemi ActiveTwo amplifier as for the EEG. The electrodes were placed on the participants’ second phalanx of the middle and ring finger of the nondominant hand after the region had been lightly cleaned with water.

**Figure 2.**
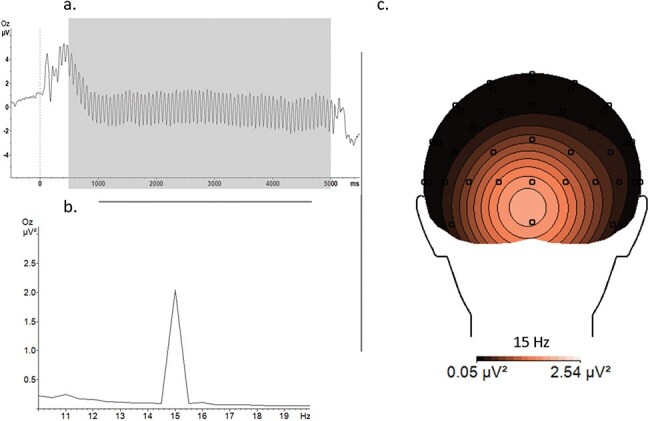
Representation of the ssVEP signal with (a) ssVEPs at the Oz electrode; the gray window marks the stimulus duration of 500–5000 ms for which the (b) FFT power of 15 Hz is extracted and shown with the (c) corresponding topography at the Oz electrode site.

BrainVizion Analyzer 2.0 (BrainProducts Inc., Gilching, Germany) was used to analyze the signal offline for both EEG and EDA. The offline filters applied for the EEG data were 0.1 Hz low cutoff, 40 Hz high cutoff, and 50-Hz notch. Ocular artifacts were detected and corrected with the Gratton-Cole artifact correction procedure (Gratton et al. 1983). Because of a technical problem with the electro-ocular electrodes, ocular artifacts were not corrected for four participants (The main analysis was conducted with and without these four participants, but there were no differences. Therefore, the results presented here are with all participants. The results without these four participants can be found in the Supplementary data.). Data were re-referenced to the average of all electrodes and segmented to a time window of −500 to 5500 ms. Artifacts were rejected according to the following criteria: (1) maximal amplitude allowed was 200 mV and (2) lowest activity allowed in intervals was 0.5 mV (100 ms interval length). The mean acceptance rate across all conditions was 96.4%. The number of remaining trials was further statistically analyzed to see if they differ per condition. There was a significant difference in the number of kept trials for the effect of Context in acquisition, CTX+: 95.5%, CTX−: 97.2%, *F*(1.48) = 55.18, *P* = .006, *η*_p_^2^ = 0.15, but the effects of Stimulus, *F*(1.48)= 0.80, *P* = .375, *η*_p_^2^ = 0.02, and the Stimulus × Context interaction, *F*(1.48)= 0.13, *P* = .725, *η*_p_^2^ = 0.003, were not significant. There were no significant differences for generalization (all *P* values > .112). The data were further averaged according to the experimental conditions, and a fast Fourier transformation (FFT) was applied to transform the signal in the frequency domain. The FFT was applied to the last 2000 ms of stimulus presentation (3000–5000 ms) to avoid any initial nonstationary ssVEP components and because conditioning effects have been shown to be more prominent there (Moratti and Keil 2005, Moratti et al. 2006, Miskovic and Keil 2013a). Based on previous studies and the topographical analysis of the signal (Fig. 2), the mean activity of electrodes Oz, Iz, O1, and O2 was further considered for the statistical analysis.

For the signal of the EDA, a high cutoff filter of 1 Hz and a low cutoff of 0.001 Hz were applied. For the analysis of the SCR, the signal was segmented in epochs of −1000 to 8000 ms after the stimulus onset. A manual foot-to-peak method was used to quantify the signal with an onset latency time window of 900–4000 ms (Boucsein 2012). As peak, we considered the first response after the foot. Responses below 0.02 μS were scored as zero. Afterward, a log transformation [log (1 + SCR)] was used to normalize the SCR distribution and the responses were averaged according to the experimental conditions. No participants were excluded based on learning performance because SCR does not constitute a direct measure of learning and excluding participants solely on this premise could lead to sample bias (Lonsdorf et al. 2019).

#### Statistical analyses

All statistical analyses were performed in RStudio (Version 4.1.2) using linear mixed models. In contrast to the registration of this study, we did not use repeated measures ANOVAs due to several limitations such as the assumption of sphericity is rarely met in conditioning studies, while linear mixed models were recommended in the field (Vanbrabant et al. 2015).

The packages used to fit the linear mixed models were lme4 and lmerTest (Bates et al. 2015, Kuznetsova et al. 2017). Significance was determined at an alpha level of 0.05 and is reported using the Kenward–Roger approximation of degrees of freedom (Kenward and Roger 1997). Each phase of the experiment was analyzed separately with SCR, ssVEPs, valence, arousal, and US expectancy as dependent variables, and participants were included as a random intercept in all models. For habituation, Stimulus (CS+/CS−) and Context (CTX+/CTX−) were entered as fixed factors. In this phase, only the ratings of valence and arousal were analyzed as there were not enough trials to analyze the physiological measures. In acquisition, the same model was used for the ratings of valence and arousal. For ssVEPs, SCR, and US expectancy, a third fixed factor Time (Acq1 for half-way through acquisition, Acq2 for the end of acquisition (For SCR and ssVEPs, Acq1 refers to the first half of acquisition and Acq2 the second half of acquisition, while for US expectancy, Acq1 refers to the offline rating half-way through the acquisition and Acq2 is at the end of acquisition.)) was entered. In the case of significant interactions, we used planned contrasts on the development of the differential stimulus effect half-way through and at the end of acquisition for CTX+ and CTX−. CS−, Acquisition 1, and CTX− were the reference levels. In generalization, the models used were the same as in habituation, but this time, the factor Stimulus had six levels (CS+, GS1, GS2, GS3, GS4, and CS−). Every stimulus/CTX combination was averaged across trials. Simple contrasts with CS− as the reference level were conducted using paired sample *t*-tests and used as a follow-up analysis for significant main effect of Stimulus. The effect sizes in the statistical models are reported as partial *R*^2^ (Jaeger et al. 2017).

In order to describe the generalization gradients as following a linear (overgeneralization) or quadratic trend, we followed the frequentist analysis with Bayesian linear models to evaluate the evidence for the two hypotheses (linear versus quadratic). We used a prespecified vector used in the previous literature (Lissek et al. 2014, McTeague et al. 2015, Ahrens et al. 2016, Stegmann et al. 2020) for each contrast as a predictor to the original models. For the quadratic trend, the weights used were +2.5334, +1.0934, −0.0267, −0.8267, −1.3067, and −1.4667, and for the linear trend, the weights used were +2.5, +1.5, +0.5, −0.5, −1.5, and −2.5 for CS+, GS1, GS2, GS3, GS4, and CS−, respectively. In the case of significant main effects or interactions including the factor Context in the frequentist analysis, the Bayesian linear models were run separately for each context. Before comparing the Bayes factors (BFs) for each trend, we evaluated the evidence for each Bayesian linear model against a random intercept model. These can be found in Table S1 of the Supplementary data. We then calculated BFs (BF_quadratic/linear_) to evaluate whether there is more evidence for a quadratic or linear trend for each measure separately, using the package BayesFactor (version 0.9.12-4.4) with default priors (Rouder et al. 2012, Jarosz and Wiley 2014). For ssVEPs, a third trend was entered in the analysis, the lateral inhibition model which was expressed as the difference between two Gaussian distributions (McTeague et al. 2015, Stegmann et al. 2020), with weights: +2, −2, +0.5, +1, +0.5, and −2. We consider BFs > 10 indicative of strong evidence for the quadratic trend and BFs < 0.10 to indicate strong evidence for the linear trend (Lee and Wagenmakers 2014). For all statistical analyses, alpha was set at 0.05 and Bonferroni correction was used for multiple testing.

Additionally, we calculated how well participants discriminated between each cue and CS+ with the discrimination task. Every cue was compared to the CS+, and participants had to answer whether the two pictures show the same face. “Yes” responses were transformed to “0,” and “No” responses were transformed to “1.” We report the average discrimination score for each cue, with numbers closer to 1 indicating better discrimination than numbers closer to 0.

Finally, as an exploratory analysis we included the questionnaires as covariates to the main models used in generalization to see if individual differences influenced the generalization gradients.

## Results

### Habituation

In habituation, neither main effects nor interactions were found to be significant for either arousal or valence (all *P* values > .207), indicating no evidence that participants differentiated between the two faces presented in each context regarding subjective arousal (CTX+: CS+: *M* = 3.79, SD = 1.61, CS−: *M* = 3.77, SD = 1.82, CTX−: CS+: *M* = 4.00, SD = 1.53, CS−: *M* = 3.83, SD = 1.67) and unpleasantness (CTX+: CS+: *M* = 4.78, SD = 1.30, CS−: *M* = 4.77, SD = 1.15, CTX−: CS+: *M* = 4.81, SD = 1.41, CS−: *M* = 4.53, SD = 1.08).

### Acquisition

#### Psychophysiology

In acquisition, the Stimulus × Context interaction for SCR [*F*(1322) = 11.80, *P *< .001, *R*^2^ = 0.035] was significant. As shown in Fig. 4, participants had greater differential response between CS+ and CS− in CTX+ (CS+: *M* = 0.07, SD = 0.08, CS−: *M* = 0.02, SD = 0.03) than in CTX− [CS+: *M* = 0.03, SD = 0.05, CS−: *M* = 0.01, SD = 0.02, *b*_Stimulus×Context_ = 0.03, SE = 0.01, *t*(326.00) = 3.44, *P* < .001]. The main effects of Stimulus [*F*(1, 322) = 65.94, *P *< .001, *R*^2^ = 0.170] and Context [*F*(1, 322) = 30.07, *P *< .001, *R*^2^ = 0.085] were also significant. However, the main effect of Time [*F*(1322) = 2.03, *P *= .155, *R*^2^ = 0.006] and all interactions including the factor Time were not significant (all *P* values > .510).

**Figure 3.**
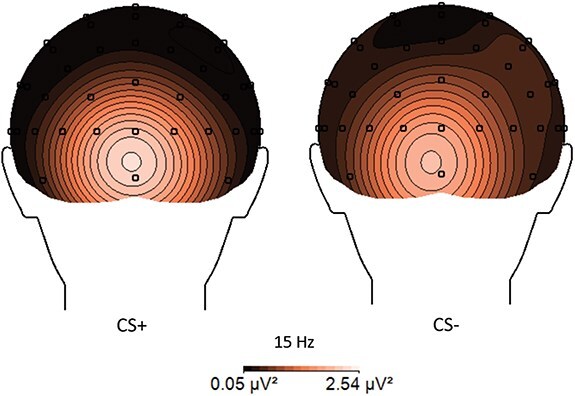
Topographical representation of the mean ssVEP amplitude for the comparison between CS+ and CS− in acquisition.

For ssVEPs, the participants showed more visuocortical response overall to CS+ (*M* = 0.87, SD = 1.67) compared to CS− (*M* = 0.75, SD = 1.08, see also Fig. 3) and overall in Acq1 (*M* = 0.95, SD = 1.68) than Acq2 (*M* = 0.77, SD = 1.22) as the main effects for Stimulus [*F*(1314.14) = 4.36, *P *= .037, *R*^2^ = 0.014] and Time [*F*(1314.14) = 7.07, *P *= .008, *R*^2^ = 0.022] were significant, respectively. The main effect of Context [*F*(1314.00) = 3.39, *P *= .066, *R*^2^ = 0.011] did not reach significance as did none of the interaction effects (all *P* values > .521). Thus, there was no evidence for preferential responding to the faces presented during CTX+ as compared to CTX− and in different parts of the acquisition.

#### Ratings

For both arousal and valence ratings, there was a significant main effect of Stimulus for arousal [*F*(1, 138) = 144.74, *P *< .001, *R*^2^ = 0.512] and valence [*F*(1138) = 149.83, *P *< .001, *R*^2^ = 0.521], with CS+ scoring more arousal [*b*_Stimulus_ = 2.63, SE = 0.22, *t*(140.00) = 12.06, *P* < .001] and more unpleasantness than CS− [*b*_Stimulus_ = 2.17, SE = 0.18, *t*(140.00) = 12.20, *P* < .001] averaged across the contexts. The main effects of Context for arousal [*F*(1138) = 0.40, *P *= .527, *R*^2^ = 0.003] and valence [*F*(1, 138) = 2.82, *P *= .095, *R*^2^ = 0.020] as well as the Stimulus × Context interaction for both arousal [*F*(1, 138) = 0.86, *P *= .356, *R*^2^ = 0.006] and valence [*F*(1, 138) = 0.06, *P *= .811, *R*^2^ = 0.000] were not significant. Means and standard deviations for the CS+ and CS− in each context can be found in Table 2.

**Table 2.** Descriptive statistics for the cues in the different contexts in acquisition for the subjective ratings.

Variable	*M* (SD)		*M* (SD)	
	CS+ in CTX+	CS− in CTX+	CS+ in CTX−	CS− in CTX−
Arousal (1–9)	7.25 (1.42)	4.43 (1.80)	6.91 (1.76)	4.49 (1.89)
Valence (1–9)	7.04 (1.57)	4.83 (1.34)	6.70 (1.78)	4.57 (1.57)
US expectancy (0–100)	66.84 (26.13)	12.90 (23.19)	51.27 (31.83)	9.21 (20.57)

**Figure 4.**
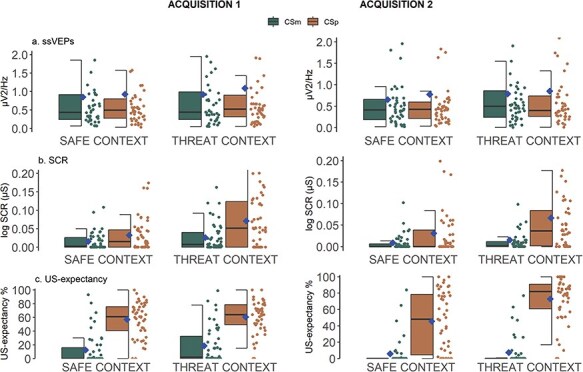
Boxplots with error bars of the conditioned responses with points for the individual responses for the measures of ssVEPs (upper panel), SCR (middle panel), and US-expectancy (lower panel) in Acquisition 1 and Acquisition 2 with diamonds indicating the means, while the middle horizontal lines show the median

For US expectancy, the linear mixed model returned a significant Stimulus × Context × Time interaction [*F*(1322) = 9.29, *P *= .002, *R*^2^ = 0.028]. When we explored this interaction separately for each Time point, we found that the Stimulus × Context interaction was significant at the end of acquisition [*F*(1138) = 13.28, *P *< .001, *R*^2^ = 0.088] but not half-way through [*F*(1, 138) = 0.11, *P *= .743, *R*^2^ = 0.001] as seen in Fig. 4. Furthermore, the Stimulus × Context interaction was significant [*F*(1, 322) = 6.68, *P *= .010, *R*^2^ = .020] with participants showing larger differential ratings to the CSs in CTX+ than CTX−. The Stimulus × Time [*F*(1322) = 4.14, *P *= .043, *R*^2^ = 0.013] and Context × Time interactions [*F*(1322) = 4.33, *P *= .038, *R*^2^ = 0.013] were also significant. Finally, the main effects of Stimulus [*F*(1322) = 435.88, *P *< .001, *R*^2^ = 0.575] and Context [*F*(1322) = 17.56, *P *< .001, *R*^2^ = 0.052] were significant. The main effect of Time did not reach significance [*F*(1322) = 3.38, *P *= .067, *R*^2^ = 0.010]]. Means and standard deviations of each stimulus in each context are displayed in Table 2.

### Generalization

#### Psychophysiology

**Figure 5.**
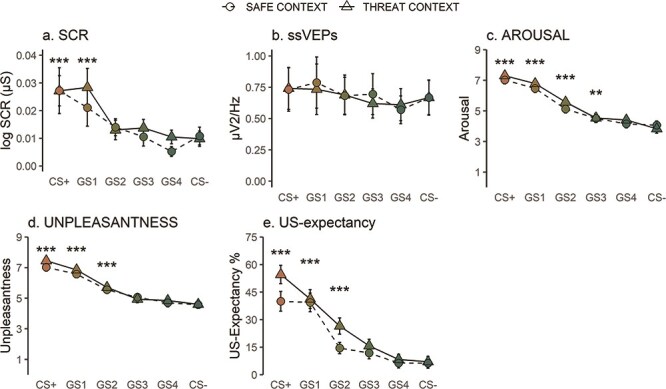
Means and standard error of the means for all test stimuli in the generalization phase. Asterisks indicate significant difference from CS− across groups.

In generalization, the linear mixed model for SCR returned a significant main effect of Stimulus [*F*(5506) = 13.82, *P *< .001, *R*^2^ = 0.120]. The main effects of Context [*F*(1506) = 1.70, *P *= .193, *R*^2^ = 0.003] and the Stimulus × Context interaction [*F*(5, 506) = 0.66, *P *= .651, *R*^2^ = 0.007] were not significant. Following up the main effect of Stimulus, we found that participants showed higher SCR for both CS+ [*b*_(CS+,CS−)_ = 0.02, SE = 0.00, *t*(512.00) = 5.53, *P* < .001] and GS1 [*b*_(GS1,CS−)_= 0.01, SE = 0.00, *t*(512.00) = 4.73, *P* < .001] compared to CS−, but the other stimuli did not differ from CS− (all *P* values > .300). Trend analysis revealed stronger evidence for the quadratic compared to the linear trend, BF_quadratic/linear_: 31.9. The generalization gradient can be seen in Fig. 5.

For ssVEPs, there was a significant main effect of Stimulus [*F*(5, 506) = 2.49, *P *= .030, *R*^2^ = 0.024], showing that there was different visuocortical responding for the different stimuli, but follow-up simple contrasts showed that none of the test stimuli differed significantly from CS− (all *P* values > .086) (Exploratorily, we followed up this effect by comparing all stimuli to each other and the results are reported in the Supplementary data.). The main effects of Context [*F*(1506) = 0.15, *P *= .703, *R*^2^ = 0.000] and the Stimulus × Context interaction [*F*(5506) = 0.32, *P *= .902, *R*^2^ = 0.003] were not significant (all *P* values > .520). With regard to the trend analysis, both linear, BF_linear/lateral inhibition_: 38.08, and quadratic trends, BF_quadratic/lateral inhibition_: 48.21, showed stronger evidence in comparison to the lateral inhibition, but there was only anecdotal evidence in favor of the quadratic trend in comparison to the linear trend, BF_quadratic/linear_: 1.26.

#### Ratings

In generalization, arousal ratings had a significant main effect of Stimulus [*F*(5506) = 105.08, *P *< .001, *R*^2^ = 0.509]. The main effect of Context [*F*(1506) = 2.96, *P *= .086, *R*^2^ = 0.006] as well as the Stimulus × Context interaction [*F*(5, 506) = 0.92, *P *= .473, *R*^2^ = 0.009] did not reach significance. Following up the main effect of Stimulus, all stimuli were compared to the CS− and the generalized responses for arousal extended to all GSs except for GS4 CS+ [*b*_(CS+,CS−_) = 3.21, SE = 0.182, *t*(512.00) = 17.62, *P* < .001], GS1 [*b*_(GS1,CS−_) = 2.68, SE = 0.182, *t*(512.00) = 14.70, *P* < .001], GS2 [*b*_(GS2,CS−)_ = 1.40, SE = 0.182, *t*(512.00) = 7.70, *P* < .001], and GS3 [*b*_(GS3,CS−)_ = 0.57, SE = 0.182, *t*(512.00) = 3.15, *P* = .002], but GS4 [*b*_(GS4,CS−)_ = 0.33, SE = 0.182, *t*(512.00) = 1.81, *P* = .071]. Trend analysis indicated very strong evidence in favor of the quadratic trend, BF_quadratic/linear_: 1.06e + 03. The generalization gradient for the arousal ratings, as seen in Fig. 5, showed a monotonic decrease from CS+ to CS−, which was slightly less steep from CS+ to GS1.

Similarly, for valence, the linear mixed model returned a significant main effect of Stimulus [*F*(5506) = 80.03, *P *< .001, *R*^2^ = 0.442] with participants reporting feeling different levels of unpleasantness in response to the test stimuli, but the main effects of Context [*F*(1506) = 2.23, *P *= .136, *R*^2^ = 0.004] as well as the Stimulus × Context interaction [*F*(5506) = 0.62, *P *= .682, *R*^2^ = 0.006] were not significant. Simple contrasts with CS− as the reference level showed that the generalization responses extended to GS2, CS+ [*b*_(CS+,CS−_) = 2.65, SE = 0.172, *t*(512.00) = 15.36, *P* < .001], GS1 [*b*_(GS1,CS−_) = 2.13, SE = 0.172, *t*(512.00) = 12.34, *P* < .001], and GS2 [*b*_(GS2,CS−_) = 1.04, SE = 0.172, *t*(512.00) = 6.05, *P* < .001]; GS3 did not survive Bonferroni correction (*a* < 0.01) [*b*_(GS3,CS−_) = 0.42, SE = 0.172, *t*(512.00) = 2.41, *P* = .016], and GS4 was not significant [*b*_(GS4,CS−_) = 0.19, SE = 0.172, *t*(512.00) = 1.11, *P* = .267]. For valence, the trend analysis revealed very strong evidence for the quadratic trend, BF_quadratic/linear_: 1.33e + 04, and the generalization gradient had a monotonic decrease from CS+ to CS− with a less steep decrease in GS1.

Finally, for US expectancy, the linear mixed model returned a significant main effect of Stimulus [*F*(5506) = 65.99, *P *< .001, *R*^2^ = 0.395], a significant main effect of Context [*F*(1506) = 11.31, *P *< .001, *R*^2^ = 0.022], and a nearly significant interaction [*F*(5506) = 1.94, *P *= .08, *R*^2^ = 0.019]. Follow-up simple contrasts for the main effect of Stimulus showed that the generalized responses extended to GS2, CS+ [*b*_(CS+,CS−_) = 40.59, SE = 3.05, *t*(512.00) = 13.31, *P* < .001], GS1 [*b*_(GS1,CS−_) = 33.61, SE = 3.05, *t*(512.00) = 11.02, *P* < .001], and GS2 [*b*_(GS2,CS−)_ = 13.84, SE = 3.05, *t*(512.00) = 4.54, *P* < .001], but GS3 did not survive Bonferroni correction [*b*_(GS3,CS−)_ = 7.12, SE = 3.05, *t*(512.00) = 2.33, *P* = .020], and GS4 was not significant [*b*_(GS4,CS−)_ = 0.63, SE = 3.05, *t*(512.00) = 0.20, *P* = .837]. Due to the significant main effect of Context in the frequentist analysis, we calculated the BFs for the two contexts separately. For both contexts, the trend analysis returned strong evidence for the quadratic compared to the linear trend, CTX+: BF_quadratic/linear_: 1.00e + 02 and CTX−: BF_quadratic/linear_: 5.73e + 01. The shape of the distributions for US expectancy for the two contexts can be found in Fig. 5.

### Discrimination task

Overall, participants discriminated well between each cue and CS+ with higher discrimination scores for GS2 (*M* = 0.81, SD = 0.39), GS3 (*M* = 0.94, SD = 0.25), GS4 (*M* = 1.00, SD = 0.00), and CS− (*M* = 0.98, SD = 0.15) and lower for GS1 (*M* = 0.38, SD = 0.49).

### Exploratory analysis

#### The relationship of individual traits and generalization

Exploratorily, we included the questionnaires as a covariate in the main analysis for generalization to see if any of the assessed predispositional factors show an influence on the generalization gradients for the two contexts. Regarding STAI-S, we found a significant Stimulus × STAI-S interaction in US expectancy [*F*(5495) = 7.23, *P *< .001, *R*^2^ = 0.068]. Follow-up contrasts revealed that there was a significant increase in US expectancy from CS− to CS+ as STAI-S scores increased by 1 unit [*b*_(CS+,STAI-S)_ = 13.07, SE = 2.96, *t*(507.00) = 4.41, *P* < .001] and for GS1 [*b*_(GS1,STAI-S)_ = 9.80, SE = 2.96, *t*(507.00) = 3.30, *P* = .001], but the change was not significant for the other stimuli (all *P* values > .555, Bonferroni correction *α* < 0.01). All other variables did not show any significant effects with STAI-S (all *P* values > .199).

When we included STAI-T in the main generalization analysis, we found significant effects for all measurements. For SCR, there was a significant Stimulus × STAI-T interaction [*F*(5495) = 3.58, *P *= .003, *R*^2^ = 0.035]. This effect was driven by a significant decrease in SCR for CS+ [*b*_(CS+,STAI-T)_ = −0.001, SE = 0.000, *t*(507.00) = 1. 97, *P* = .049] and GS1 [*b*_(GS1,STAI-T)_ = −0.002, SE = 0.000, *t*(507.00) = −2. 47, *P* = .014] as STAI-T scores rise by 1 unit. However, none of these relationships survived Bonferroni correction (*α* < 0.01). For ssVEPs, there was a significant Stimulus × Context × STAI-T interaction [*F*(5495) = 2.65, *P *= .022, *R*^2^ = 0.026]. We followed up this interaction with separate analyses for each context. In both CTX+ and CTX−, 1 unit of increase in STAI-T was associated with higher visuocortical responding to GS1 than CS−: CTX+ [*b*_(GS1,STAI-T)_ = 0.29, SE = 0.07, *t*(225.00) = 4.12, *P* < .001], CTX− [*b*_(GS1,STAI-T)_ = 0.24, SE = 0.08, *t*(225.00) = 3.10, *P* = .002]. All other comparisons did not survive Bonferroni correction. For the subjective ratings, there was a significant Stimulus × STAI-T interaction as well: US expectancy [*F*(5, 495) = 4.15, *P *= .001, *R*^2^ = 0.040], arousal [*F*(5, 495) = 6.58, *P *< .001, *R*^2^ = 0.065], and valence [*F*(5, 495) = 3.99, *P *= .001, *R*^2^ = 0.039]. This interaction was driven by a significant increase for CS+ and GS1; US-expectancy: CS+ [*b*_(CS+,STAI-T)_ = 1.80, SE = 0.638, *t*(507.00) = 3.51, *P* = .005] and GS1 [*b*_(GS1,STAI-T)_ = 2.24, SE = 0.638, *t*(507.00) = 3.51, *P* < .001], but GS2 did not survive Bonferroni correction [*b*_(GS2,STAI-T)_ = 1.28, SE = 0.638, *t*(507.00) = 2.09, *P* = .045], and arousal: CS+ [*b*_(CS+,STAI-T)_ = 0.13, SE = 0.038, *t*(506.99) = 3.42, *P* < .001] and GS1 [*b*_(GS1,STAI-T)_ = 0.15, SE = 0.038, *t*(506.99) = 3.94, *P* < .001], but GS2 did not survive Bonferroni correction [*b*_(GS2,STAI-T)_ = 0.08, SE = 0.038, *t*(506.99) = 2.11, *P* = .036]. For valence ratings, neither CS+ nor GS1 survived Bonferroni correction (all *P* values > .013, Bonferroni correction *α* < 0.01).

Regarding BDI, there was a significant Context × BDI interaction in valence ratings [*F*(5495) = 4.58, *P *= .032, *R*^2^ = 0.009]. However, follow-up contrasts showed that the increase for valence ratings to the cues in CTX+ was not significant [*b*_(CTX+,BDI)_ = 0.21, SE = 0.132, *t*(515.00) = 1.61, *P* = .108]. None of the other measures showed significant effects with BDI (all *P* values > .106).

For IUS, there was a significant Stimulus × IUS interaction for US-expectancy ratings [*F*(5495) = 3.21, *P *= .007, *R*^2^ = 0.031], which was driven by an increase for GS1 [*b*_(GS1,IUS)_ = 0.54, SE = 0.166, *t*(507.00) = 3.25, *P* = .001] and GS2 [*b*_(GS2,IUS)_ = 0.39, SE = 0.166, *t*(507.00) = 2.33, *P* = .020]; however, GS2 did not survive Bonferroni correction (*α* < 0.01). No other significant effects were found with IUS and the other measures (all *P* values > .199).

Finally, regarding LSAS, we did not find any significant effects for any of the measures (all *P* values > .403).

## Discussion

The goal of this study was to investigate whether a threatening context increases the width of cue generalization using different psychophysiological and subjective measures. As per the first goal and in contrast to our expectations and previous literature (Klein et al. 2021), our study found no evidence that threatening context increases the width of cue generalization in any of the measured variables. We, however, observed that threatening context increases the overall threat expectancies compared to the safe context.

These findings point to successful learning of the context contingencies and an adaptive response to a context where threat is likely to occur. Our participants seemed to understand that the US will occur after the CS+ only in the threatening context and they expected the US less as stimuli similarity decreased from the CS+ in an adaptive manner (both contexts showed a quadratic generalization gradient) (Lissek et al. 2008, Nelson et al. 2015, Ahrens et al. 2016, Kaczkurkin et al. 2017). However, despite correct contingency awareness of the two contexts, the threatening context did not seem to affect participants’ preferential visuocortical responding, autonomic arousal, and their view of the different cues as unpleasant or arousing. Furthermore, threatening context did not seem to increase the width of cue generalization.

Several factors could have influenced the observed results. First, it could be that cue conditioning overrode the effects of context. Cue conditioning in our study was successful for both subjective and physiological measures, as participants learned the difference between the CSs in both contexts. However, the responses to the cues did not differ according to context in all measures (ssVEPs, affective ratings). Although the same type of context has been used successfully previously (Wieser et al. 2016b, Stegmann et al. 2019), these studies used a different experimental design where participants were instructed about the contextual differences. Additionally, in the study by Klein et al. (2021), two different rooms constituted the contexts, which are more distinguishable than the contexts chosen in our study (i.e. geometrical shapes) as there are more stimuli present that could signal the difference in the context between CTX+ and CTX−. On the contrary, the contexts in our study included fewer stimuli (only the geometrical shapes) which are perceptually fairly similar and can be easily confused. This perceptual similarity of the contextual cues might have led to context generalization in both acquisition and generalization phases, similar to the study by Andreatta et al. (2020) as the CSs in the two contexts had similar responses in the affective ratings, ssVEPs, and SCR (only in generalization). Two other studies indeed suggest that contexts that have to be learned may seem to be less threatening in a cue-in-context conditioning paradigm (Stegmann et al. 2024), whereas inherently threatening contexts such as aversive pictures indeed are able to influence visuocortical and psychophysiological conditioned threat responses (Stegmann et al. 2023).

Secondly, the participants were well-functioning university students, and their anxiety levels might not be debilitating enough to show cue overgeneralization. Although the mean trait anxiety level in our study was above the suggested threshold (>40; see Table 1; Addolorato et al. 1999), other studies suggest that a higher cutoff is more specific to clinical anxiety (Kvaal et al. 2005, Julian 2011). These results agree with the literature suggesting that fear overgeneralization can have a diagnostic effect for clinical anxiety. The discussion regarding the presence of overgeneralization in subclinical anxiety levels is still ongoing with a small positive correlation found in the latest meta-analyses (Sep et al. 2019, Cooper et al. 2022). In our study, we found significant associations with STAI-T and how participants responded to GS1 in all measured variables, which suggests the presence of an association. However, it was not enough for participants to show cue overgeneralization in the threatening context compared to the safe one.

Another point of interest that could have influenced the current findings is the order of CTX trials in acquisition. In our study, CTX+ was presented first for all participants, in order to boost their learning of the context contingencies as we did not provide any instructions about it. This meant that participants initially learned the associations between the CSs and US in CTX+ and then they had to learn the conditional contingencies of the CSs with the US in CTX−. In CTX−, participants had to learn to inhibit the responses to the CS+ as a second rule to the CS+/US association learned previously (Bouton 2002) and might have therefore needed more trials to learn it as inhibition of a conditioned response highly depends on the amount of trials presented (Bouton 2004). Although the number of trials used in the current study was not less than the ones used in previous studies (Klein et al. 2021), it might have been insufficient for participants to fully comprehend the contingencies of the two contexts with the US as well as inhibit the fear responses during CTX− especially since they did not receive any instructions on the context differences. Consequently, more salient contexts including more stimuli such as different rooms (Kastner et al. 2015, Wieser et al. 2016b, Klein et al. 2021) as well as more context trials could lead to better discrimination between the contexts and therefore, potentially, a greater difference in the width of the cue generalization.

A second aim of this study was to investigate how cue-in-context learning and generalization are expressed in the different psychophysiological measures and subjective ratings. Interestingly, a different time course of skin conductance and US expectancy emerged during acquisition. Specifically, differences in the responses to cues in the different contexts were captured in SCR even after one presentation of each context, while this difference was observed in US expectancy only after the entire acquisition phase. Despite the fact that both measures are considered to represent measures of cognitive awareness (Dawson and Biferno 1973, Ross and Nelson 1973, Biferno and Dawson 1977) and are well-established measures of associative learning (Dawson et al. 2007, Lonsdorf et al. 2017), they measure defensive responses at different levels. More specifically, SCR, as a measure of autonomic arousal, reflects the motivational relevance of a stimulus and is a fast response to contingency changes in fear conditioning (Lang et al. 1993, Cacciaglia et al. 2015, Constantinou et al. 2021). On the other hand, US expectancy, similar to other verbal ratings, represents a top-down response on the CS-US contingencies, which relies on working and declarative memory processes (Carter et al. 2006, Boddez et al. 2013, Cacciaglia et al. 2015). Additionally, the two measures seem to be mediated by different brain structures. Specifically, the magnitude of SCRs to conditioned stimuli is mediated by amygdalar volume (Cacciaglia et al. 2015) and activity (Carter et al. 2006) during acquisition of fear, especially during early trials. On the other hand, US-expectancy ratings are mediated by hippocampus as well as middle frontal gyrus (Carter et al. 2006, Cacciaglia et al. 2015), which implicates the role of working memory in expectancy ratings. The difference in the timeline of these two responses in our study adds to the literature on the differences between implicit and explicit contingency awareness (Büchel et al. 1998, LeDoux 2003). Specifically, our results suggest that implicit awareness of fear contingencies, as reflected by SCRs, precedes explicit awareness as measured by US-expectancy ratings and the time difference between the two measures in acquisition could reflect the time it takes for the verbal fear memory to be developed and processed (van Kesteren et al. 2010). Of crucial importance, this pattern was not maintained in fear generalization. On the contrary, SCRs to the cues did not differ between the contexts, while US-expectancy ratings were increased overall in the threatening context. These results are in line with the hypothesis that SCR is involved in the initial stages of fear learning rather than the expression or retention of fear (Cacciaglia et al. 2015) and its role could therefore be to inform other levels of threat responding such as threat expectancy and selective visual attention (Moratti et al. 2006).

Another important divergence in the measures in this study was observed in the different subjective ratings. While US expectancy demonstrated a later (the difference emerged only after the entire acquisition phase) but sustained difference in the cues in the different contexts, we found no evidence that participants differentiated between the cues presented in the different contexts in the affective ratings. In the literature, the relationship between different verbal reports of associative (e.g. US expectancy) and evaluative (e.g. affective ratings) conditioning remains elusive. Some studies suggest that the (un)pleasantness and arousal of the CS cue are not modulated by contingency awareness (Baeyens et al. 1993) and remain unaffected by inhibitory learning procedures such as extinction (Diaz et al. 2005, Luck and Lipp 2015) both immediately after acquisition and at the 2-month follow-up (Baeyens et al. 1988). However, other studies show that persistent threat expectancies during extinction are predictive of negative affect at the end of extinction (Constantinou et al. 2021). These associations, although not consistent in the literature, bare great clinical relevance and have been reported to be indicative of return of fear (Dirikx et al. 2004, 2007). Our results add to the literature on the differences between associative and evaluative conditioning. However, in other studies using different contexts, participants were able to differentiate between safe and threatening context in terms of valence and arousal (Kastner et al. 2015, Kastner-Dorn et al. 2018) but showed resistance to extinction. Therefore, an alternative explanation could be that participants needed more context trials to show differences between the cues in the different contexts in the affective ratings as evaluative conditioning tends to be slower in inhibitory learning. Additionally, in the current study, the affective ratings were assessed with questions about the cues presented in the different contexts, not about the contexts themselves. Therefore, participants found the cues equally arousing and unpleasant regardless of the surrounding contexts.

Regarding ssVEPs, participants showed preferential visuocortical responding of the CS+ in acquisition but no evidence for contextual influence on this cue responding. According to the literature, despite clear and sustained differences in the visuocortical processing of safe and threatening contexts (Kastner et al. 2015, Wieser et al. 2016b, Kastner-Dorn et al. 2018) this is not always the case regarding the cues presented in safe and threatening contexts (Kastner-Dorn et al. 2018, Stegmann et al. 2019). Furthermore, contrary to our expectations and previous literature (McTeague et al. 2015, Antov et al. 2020, Stegmann et al. 2020), we could not replicate the findings of lateral inhibition in cue generalization gradients. One reason could be that for this pattern to emerge, the GSs need to be presented already in acquisition, as was done in McTeague et al. (2015) and Antov et al. (2020), as it could enhance the discrimination between CS+ and other stimuli.

Interestingly, the ssVEP results matched more with the results from the affective ratings rather than the other psychophysiological measure, SCR. A dissociation between sensory and peripheral measures has been found before (McTeague et al. 2015, Stegmann et al. 2020). In general, ssVEPs as well as steady-state visual evoked fields (ssVEFs, their magnetoencephalographic counterpart) reflect stimulus-driven neural oscillations in the early visual cortex (Moratti and Keil 2005, Wieser et al. 2016a). However, contingency awareness alone does not seem to drive ssVEPs/ssVEFs (as does for SCR) but rather the motivational relevance of the stimulus (such as fear) (Moratti and Keil 2005, Moratti et al. 2006). It has been suggested that these short-term changes in the visual cortex that enhance visuocortical processing of threat-related stimuli might be induced by anterior as well as subcortical projections such as the amygdala (Damasio 1998, Davis 1998, Miskovic and Keil 2012, 2013a) possibly exerting top-down influences. Therefore, peripheral responses such as SCR might reflect faster responses in response to contingency changes in fear conditioning paradigms, while ssVEPs might represent re-entrant signals with top-down influences from anterior areas driven by the threat-induced changes. In fact, peripheral autonomic responses such as heart rate have been shown to drive the ssVEF signal (Moratti and Keil 2005, Moratti et al. 2006).

The differences observed between the measures in this study might reflect an evolutionary mechanism that supports flexible and efficient threat detection and responding in a dynamic and ever-changing environment. Responses to danger are varied because danger itself varies (Fanselow and Pennington 2018). By this account, autonomic arousal would reflect threat detection in a fast manner, responding to the motivational relevance of the stimulus and potentially driving other levels of threat detection such as ssVEPs (Moratti and Keil 2005, Moratti et al. 2006) in order to promote visual facilitation of the threatening stimulus. Expectancy of threat operates on a second level reflecting top-down explicit awareness of the threat but not necessarily the affective aspects. Additionally, the expectancy of threat might drive the affective responses on a third level (Constantinou et al. 2021). The product of the outcomes of all these systems could be the mechanism behind an individual response in a given threatening situation. Interestingly, it makes sense that the systems involved in the early detection of threat (SCR, ssVEPs) are more malleable to change and sensitive to sensory aspects of the threat in order to promote flexibility, while others such as the affective ratings are less malleable in order to prevent costly misses. Additionally, this difficulty in changing the affective aspects of a threat might reflect the difficulty in treating clinical anxiety. Importantly, these, less flexible, systems also often show even less flexibility in clinical samples such as in post-traumatic stress disorder (PTSD) (Steiger et al. 2015) and can predict the return of fear (Dirikx et al. 2004, 2007). From this view, fear is a multidimensional response to danger for which many systems need to coordinate and none of them can fully explain fear independently (Barrett 2017, Fanselow and Pennington 2018).

Finally, in agreement with previous studies, we found an association between how participants responded to the stimuli in generalization in subjective ratings (US-expectancy and arousal) with higher trait anxiety (Haddad et al. 2012, Klein et al. 2021, Wong and Lovibond 2018). More specifically, higher US-expectancy and arousal ratings were found for CS+ and GS1. These results add to the literature showing that people scoring high on anxious personality traits tend to generalize fear more (Sep et al. 2019, Cooper et al. 2022). Our study is also among the first to show an association between trait anxiety and visuocortical responding in generalization. Additionally, we replicated previous findings showing an association between fear generalization and intolerance of uncertainty (Aslanidou et al. 2023), as we found higher threat expectancies to GS1 with higher intolerance of uncertainty. However, similar to previous studies (Andreatta et al. 2020), there was no evidence that contextual information influenced these associations. More research is needed to determine the mechanisms that drive these inconsistencies.

This study has several limitations that need to be addressed. One limitation is the duration of the cue-in-context presentations before the affective ratings were taken as compared to the duration in the experimental phases. The presentation of the cue-in-context trials lasted for only 1 s in contrast to the trials of the US-expectancy ratings in which the stimuli were present on the screen until participants gave a response. This duration could have resulted in too little time for participants to discern the context differences and could have influenced the lack of context differences in the affective ratings. Another limitation is the reinforcement schedule in the generalization phase. During generalization, the CS+ was reinforced 20% of the time in the CTX+ in line with previous studies in our laboratory (Aslanidou et al. 2023) in order to promote the emergence of individual differences (Lissek et al. 2006). However, this change in the reinforcement schedule from acquisition to generalization is different from the reinforcement changes in other generalization studies (Lissek et al. 2008, Dunsmoor et al. 2017) and could have therefore influenced cue generalization. Furthermore, the generalization test took place immediately after acquisition, which could have aided in cue discrimination (Onat and Büchel 2015, Kausche et al. 2021). In fact, testing generalization on a subsequent day is a more realistic approach and has been shown to increase the width of the generalization gradient (Kausche et al. 2021). This influence of time in the width of the cue generalization was explained by the authors by a lack of specificity of the fear memory regarding the differences between CS+ and the cues most similar to it. In the current study, generalization was tested in 1 day. It is therefore not surprising that the shape of the generalization gradients remained in adaptive levels (quadratic). However, it would be interesting if the increased threat expectancies in the threatening context observed in our study had a top-down influence on fear generalization 24 h later. This could point to an intermediate step in forming a maladaptive response and could inform our understanding of the mechanisms that modulate generalization after successful fear learning. Additionally, the ISI chosen in this study is shorter than that recommended for SCR (Lonsdorf et al. 2017). We countered this shortcoming by analyzing the SCR manually and trials that did not reach baseline before the stimulus presentations were marked as 0. However, this could have led to a greater number of nonresponders. Finally, regarding the ssVEPs, there were more artifacts and consequently rejected CTX+ trials than in CTX−. This asymmetry could reflect an effect of context conditioning, but it could also be due to increased muscle movement caused by the presence of the US. In our study, the US trials were not marked with a trigger, and therefore we cannot safely make this conclusion. However, future studies should consider using US-free only trials.

Taken together, in this study, participants demonstrated successful fear learning and adaptive generalization gradients in both subjective and psychophysiological measures of defensive responding. Higher responses were observed for cues that resembled the fear cue but decreased rapidly as similarity also decreased. Contextual information did not seem to modulate the width of fear generalization in response to the different cues. Additionally, trait anxiety was associated with participants’ responses toward the GSs that resembled the threatening one. This study also provides evidence regarding the different roles and time course of the psychophysiological and cognitive-emotional systems involved in threat detection and fear generalization. However, we found no evidence for the lateral inhibition model of visuocortical responding in generalization.

## Supplementary Material

nsae097_Supp
