# Supplementary material for: Generalized expectancy of threat in threatening compared to safe contexts
Source: Soc Cogn Affect Neurosci. 2024 Dec 16;20(1):nsae097. doi: 10.1093/scan/nsae097 (PMC11711681; doi:10.1093/scan/nsae097)
Supplement: nsae097_Supp [file nsae097_supp.zip › scan-24-171-File009.docx]

| **Table S1**  *Scientific notations for Bayes factors obtained for the basic Bayesian linear model against the random intercept model for each trend tested.* | | | |
| --- | --- | --- | --- |
| Variable | Quadratic trend | Linear trend | Lateral inhibition trend |
| SCR | 4.40e+64 | 2.16e+77 | - |
| ssVEPs | 2.36e+211 | 1.187e+211 | 4.91e+209 |
| Arousal | 7.71e+101 | 6.96e+98 | - |
| Valence | 6.86e+33 | 2.68e+93 | - |
| US-expectancy |  |  |  |
| CTX+ | 2.43e+6 | 1.53e+34 | - |
| CTX- | 2.73e+6 | 2.70e+27 | - |

***Analysis on ssVEPs excluding the participants without ocular correction***

In acquisition the main effects of Stimulus [*F*(1, 294) = 5.26, *p* = .022, *R2* = .018] and Time [*F*(1, 294) = 7.92, *p* = .005, *R2* = .026] was significant while Context [*F*(1, 294) = 3.13, *p* = .078, *R2* = .011] and the interactions were not significant (all *p* values > .604). Similarly, in generalization the main effect of Stimulus was significant [*F*(5, 462) = 2.66, *p* = .022, *R2* = .028] while all other effects were not (all *p* values > .608).

***Follow-up analysis on the main effect of Stimulus on ssVEPs in generalization***

When we explored the data further for this effect we found that the difference lies in GS4 (so the GS closest to CS-) when compared to CS+ [*b*(CS+, GS4) = 0.15, *SE* = 0.05, *t*(512.00) = 2.71, *p* = .007] and GS1 [*b*(GS1, GS4) = 0.17, *SE* = 0.05, *t*(512.00) = 3.15, *p* = .001].

***Main analyses excluding males***

*Acquisition*

*SCR,* the Stimulus x Context interaction remained significant [*F*(1, 252) = 9.69, *p* < .001, *R2* = .037] as did the separate main effects of Stimulus [*F*(1, 252) = 48.05, *p* < .001, *R2* = .160] and Context [*F*(1, 252) = 31.33, *p* < .001, *R2* = .111].

*ssVEPs,* the main effects of Stimulus [*F*(1, 244.14) = 4.89, *p* = .028, *R2* = .020] and Time [*F*(1, 244.14) = 5.89, *p* = .016, *R2* = .024] remained significant while all other effects not (all *p* values > .066).

*Arousal,* the main effect of Stimulus remained significant [*F*(1, 108) = 107.37, *p* < .001, *R2* = .499] but no other effects did (all *p* values >.711).

*Valence,* the main effect of Stimulus remained significant [*F*(1, 108) = 119.32, *p* < .001, *R2* = .525] but no other effects did (all *p* values >.135).

*Expectancy,* the Stimulus x Context x Time interaction remained significant [*F*(1, 252) = 4.62, *p* = .032, *R2* = .018].

*Generalization*

*SCR,* the main effect of Stimulus remained significant [*F*(5, 396) = 9.13, *p* < .001, *R2* = .103] *but no other effects did* (all *p* values >.107)*.*

*ssVEPs,* the main effect of Stimulus remained significant [*F*(5, 396) = 2.53, *p* = .029, *R2* = .031] while all other effects not (all *p* values > .645).

*Arousal,* the main effect of Stimulus remained significant [*F*(5, 396) = 97.02, *p* < .001, *R2* = .551] but no other effects did (all *p* values >.417).

*Valence,* the main effect of Stimulus remained significant [*F*(5, 396) = 67.43, *p* < .001, *R2* = .460] but no other effects did (all *p* values >.474).

*Expectancy,* there was a significant main effect of Stimulus [*F*(5, 396) = 57.03, *p* < .001, *R2* = .426] but the main effect of Context [*F*(1, 396) = 3.57, *p* = .059, *R2* = .009] did not reach significance neither did the interaction [*F*(5, 396) = 1.14, *p* = .337, *R2* = .014].
